# Biological Liquid–Liquid Phase Separation, Biomolecular Condensates, and Membraneless Organelles: Now You See Me, Now You Don’t

**DOI:** 10.3390/ijms241713150

**Published:** 2023-08-24

**Authors:** Vladimir N. Uversky

**Affiliations:** Department of Molecular Medicine and Byrd Alzheimer’s Research Institute, Morsani College of Medicine, University of South Florida, Tampa, FL 33612, USA; vuversky@usf.edu

Liquid–liquid phase separation (LLPS, also known as biomolecular condensation) and the related biogenesis of various membraneless organelles (MLOs) and biomolecular condensates (BMCs) are now considered fundamental molecular mechanisms governing the spatiotemporal organization of the intracellular space [1,2,3,4,5]. Physically, LLPS represents a special case of phase transition taking place in a homogeneous solution, i.e., a system including a solvent (in this case, water) and homogenously distributed solutes (e.g., proteins and nucleic acids). Under specific conditions, this homogeneous solution undergoes a process of spontaneous separation into two (or more) distinct immiscible liquids reflected in the emergence of dense and dilute phases that, respectively, contain more and less specific solutes. Although as a result of this phase transition, the chemical nature of solvent is not changed, and dense and dilute phases are still filled with water, the resulting separated phases are characterized by noticeable changes in the solvent properties of water [6]. Therefore, in addition to the consideration of the peculiarities of the interaction between different solutes undergoing LLPS, one should keep in mind that water plays a crucial role in biological LLPS and in the biogenesis of various MLOs [6].

When LLPS of biopolymers takes place in a cell, it leads to the emergence of MLOs or BMCs, which, being liquid droplets, represent specific compartments within a cell that are not enclosed by a lipid membrane [7,8,9,10]. Therefore, the biogenesis of MLOs is linked to the ability of biopolymers (e.g., intrinsically disordered proteins (IDPs) [11,12,13] and nucleic acids [14,15]) to separate into phases under specific conditions. LLPS is known to be controlled by various external factors and environmental cues, such as changes in the temperature, pH, ionic strength of the solution, posttranslational modifications [16], small molecules [17], and a number of other causes [18]. Furthermore, most LLPS processes are reversible, and many MLOs exist transiently and show “now you see me, now you don’t” behavior, rapidly emerging when conditions are changed and rapidly disintegrating as soon as the original conditions are restored.

MLOs/BMCs are many (to date, about a hundred different MLOs are known [19]) and can be found in eukaryotes, archaea, bacteria, and, likely, viruses. They exist as liquid droplets (or cellular bodies, puncta, etc.) in the cytoplasm, nucleoplasm, mitochondrial matrix, and stroma of chloroplasts. Figure 1 displays the dramatic increase in the appreciation of the importance of MLOs in recent literature. Despite being almost unknown to researchers until quite recently, MLOs are rapidly becoming mainstream in modern cellular research. In line with this ever-increasing interest in MLOs, this Special Issue includes seven research articles and three reviews considering different aspects related to the LLPS, MLOs, and BMCs. These articles are briefly outlined below.

In their research article, Mohtadin Hashemi, Siddhartha Banerjee, and Yuri L. Lyubchenko investigated the effects of membrane and free cholesterol on the early stages of the aggregation process of amyloid β (Aβ) [20]. The authors showed that the aggregation of this protein, which is related to the formation of neurotoxic species in Alzheimer’s disease, is dramatically enhanced via direct Aβ-membrane interactions. Importantly, this interaction promotes oligomer assembly on the lipid bilayer at physiologically low concentrations of the Aβ monomer. The process is strongly dependent on the membrane composition, and the presence of cholesterol in the membranes significantly enhances the aggregation kinetics. Furthermore, in agreement with previous studies where the presence of free cholesterol in amyloid plaques was reported, the authors found that free cholesterol can further accelerate the aggregation process and promote fast formation of aggregates of significantly larger sizes (as evidenced by an in-solution time-lapse Atomic Force Microscopy (AFM) analysis). Furthermore, free cholesterol accelerated the dissociation of Aβ oligomers from the surface and their accumulation in bulk solution [20].

Juliet F. Nilsson, Hakima Baroudi, Frank Gondelaud, Giulia Pesce, Christophe Bignon, Denis Ptchelkine, Joseph Chamieh, Hervé Cottet, Andrey V. Kajava, and Sonia Longhi also studied the formation of amyloid fibrils, but analyzed the aggregation behavior of an interesting viral protein, the phosphoprotein (P protein) from the Nipah and Hendra viruses (NiV and HeV), which is an essential polymerase cofactor [21]. A peculiar feature of this system is that the gene encoding the P protein in both NiV and HeV also encodes the V and W proteins. These P, V, and W proteins have unique C-terminal domains (CTD) but share an intrinsically disordered N-terminal domain (NTD). A short PTN3 region found within the shared NTD is capable of amyloid-like structure formation. The HeV PNT3 containing the amyloidogenic motif (EYYY) was used to analyze the relevance of each of three contiguous tyrosine residues to the fibrillation process. This analysis revealed that the ability to form fibrils is dramatically reduced by the removal of a single tyrosine independently of its position. Furthermore, the authors showed that PNT3 fibrillation can be regulated by the C-terminal half of this protein, showing an inhibitory effect on fibril formation [21].

Aleksandra E. Badaczewska-Dawid, Vladimir N. Uversky, and Davit A. Potoyan reported the development of a convenient web platform, Bioinformatics Analysis of LLPS Sequences (BIAPSS) [22]. The need for such a tool is based on the premises that despite a broad acceptance of the importance of biological LLPS, MLOs, and BMCs, there is a remarkable gap in the current knowledge which prevents a complete understanding of the sequence “codes” of phase separation required for the design of new phase-separating sequences of fundamental, medical, and technological importance. Therefore, the goals of this tool were to enhance apprehension of the interplay between the primary sequences of proteins and their capability to undergo spontaneous LLPS and thereby to uncover the sequence-encoded signals of the LLPS potential and related biogenesis of numerous functional MLOs and cellular bodies [22]. Researchers can use this web server in on-the-fly analysis as BIAPSS provides a useful tool for the visualization and interpretation of the physicochemical and structural features for the superset of curated LLPS proteins [22].

Yoon-Jeong Choi, Yujin Lee, Yuxi Lin, Yunseok Heo, Young-Ho Lee, and Kiwon Song dedicated their research article to the investigation of a P-body-associated protein from *Saccharomyces cerevisiae*, Nst1 [23]. The authors emphasized that not all highly promiscuous proteins found within MLOs are made equal, with only a few of them acting as key scaffolds. Being capable of inducing condensation of the components of P-bodies (PBs), the multidomain scaffolding protein Nst1 serves as an important subject for targeted analysis of the roles of different domains in biomolecular condensation processes. To this end, a series of Nst1 domain deletion mutations were prepared and investigated [23]. This analysis revealed several important features. For example, deletion of the aggregation-prone domain (APD) significantly inhibited self-condensation, whereas the Nst1 mutant with the deleted multivalent polyampholyte domain (PD) within the intrinsically disordered region (Nst1_∆PD_) was able to form self-condensates but failed to interact and condensate Dcp2, a decapping protein and PB component. Furthermore, the Nst1_∆PD_ deletion mutant was also unable to condensate other PB components, such as Xrn1, Dhh1, and Edc3, indicating that the PD of the IDR in Nst1 functions as a hub domain interacting with other PB components [23].

The research team of Tamami Miyagi, Rio Yamazaki, Koji Ueda, Satoshi Narumi, Yuhei Hayamizu, Hiroshi Uji-i, Masahiko Kuroda, and Kohsuke Kanekura investigated how the patterns of charged amino acids and the net charge within proteins undergoing LLPS determine their targeting of the specific MLOs, such as nuclear speckles and the nucleolus [24]. This was an important exercise aimed at understanding the basic mechanisms underlying the distributions of the LLPS-prone proteins with charged low-complexity domains (LCDs) to specific MLOs. The authors used proteins with Arg-enriched mixed-charge domains (R-MCDs) primarily composed of R and Asp (D) and known to accumulate in nuclear speckles via LLPS. They demonstrated that the distribution of R-MCD can be shifted from nuclear speckles to the nucleolus by redistributing their R and D residues from an alternately sequenced pattern to uneven blocky sequences [24]. Furthermore, it was established that the blocky R-MCD peptide showed affinity to RNA, acidic poly-Glu, and the acidic nucleolar protein nucleophosmin and was capable of efficient phase separation. On the other hand, the R-MCD peptide with alternating amino acids did not undergo LLPS. Furthermore, localization of the R-MCDs to the MLOs and their accumulation in the nucleolus were promoted by the incorporation of the basic residues into the R-MCDs. Based on these observations, it was concluded that the proximal positioning of D and R linked to the mutual neutralization of their charges is required for the distribution of proteins to nuclear speckles [24].

A series of two papers from the group headed by Ji-Long Liu is dedicated to the analysis of cytoophidia, filamentous structures formed by the CTP synthase (CTPS) [25,26]. Cytoophidia, or “cellular snakes”, are evolutionary conserved MLOs found in the cells of many species in all three domains of life and represent an interesting case of metabolic regulation via enzyme filamentation and resulting compartmentalization [27,28]. In the first paper of this miniseries, Yi-Fan Fang, Yi-Lan Li, Xiao-Ming Li, and Ji-Long Liu used fluorescence recovery after photobleaching (FRAP) to study the dynamic characteristics of cytoophidium in human cell lines and also utilized stimulated emission depletion (STED) microscopy to analyze the super-resolution structure of these cellular snakes [25]. This analysis revealed that cytoophidia are dynamic and reticular, with the reticular structure of CTPS cytoophidia potentially providing space for other components [25], such as inosine monophosphate dehydrogenase (IMPDH), another enzyme capable of cytoophidia formation [29]. Curiously, in their structural analysis, the authors also observed CTPS granules with tentacles [25].

In the second paper of the miniseries, Qiao-Qi Wang, Dong-Dong You, and Ji-Long Liu used the female reproductive system of Drosophila as a model for studying the physiological function of cytoophidia [26]. Utilization of a CTPSH355A mutant with a diminished cytoophidium-forming ability revealed the ingression and increased heterogeneity of follicle cells in the CTPSH355A egg chambers, indicating that cytoophidia may play a role in upholding the integrity of the follicle epithelium [26].

This Special Issue concludes with three interesting reviews regarding important biological implementations of LLPS. Lin Zhang, Shubo Wang, Wenmeng Wang, Jinming Shi, Daniel B. Stovall, Dangdang Li, and Guangchao Sui considered the associations between aberrant LLPS, misbehaving MLOs, and the development of various pathological conditions [30]. The authors systemized the properties of different MLOs and BMCs and summarized the multiple LLPS-regulated biological processes. They also emphasized that although normally functioning LLPS controls the biogenesis and composition of dozens of MLOs and BMCs in the cell, the onset and progression of various diseases, including neurodegenerative disorders and cancers, may be associated with (or even driven by) altered physiological conditions or genetic mutations, because of which phase-separated condensates may undergo aberrant formation, maturation, or gelation [30].

Woei Shyuan Ng, Hendrik Sielaff, and Ziqing Winston Zhao focused their review on phase-separation-mediated chromatin organization and dynamics [31]. The authors show how cells can utilize specific physico-chemical properties of chromatin-based phase condensates with various regulatory functions in a spatially and temporally controlled manner. In addition to presenting some key recent findings on the mechanistic roles of phase separation in regulating the organization and dynamics of chromatin-based molecular processes and illuminating the complex phase-separation-mediated interplay between chromatin and diverse chromatin-interacting molecular species, the authors also focused on the quantitative characterizations of these condensates using advanced imaging-based approaches. They emphasized that such phase-separation-mediated chromatin organization defines an emerging multifaceted, multimodal, and multiscale landscape responsible for the hierarchical regulation of the genome. The authors also discussed some deficiencies in existing studies and emphasized the need for multiparametric approaches for the in-depth characterization of chromatin-based phase separation in close-to-native cellular contexts [31].

Finally, Olga I. Povarova, Iuliia A. Antifeeva, Alexander V. Fonin, Konstantin K. Turoverov, and Irina M. Kuznetsova discussed the important roles of LLPS in the spatiotemporal regulation of the cytoskeleton assembly/disassembly, including the formation of actin filaments [32]. The authors argued that the LLPS leading to the formation of coacervates of actin-binding proteins can increase local concentration of G-actin and thereby initiate its polymerization. Furthermore, coacervates can acts as biological reactors, wherein, in addition to this local increase in the G-actin concentration, integration of proteins controlling actin polymerization, such as N-WASP, Arp2/3, and Cortactin, into such MLOs enhances the activity of these actin-binding proteins, thereby providing additional means for the efficient formation of actin filaments [32].

## Figures and Tables

**Figure 1:**
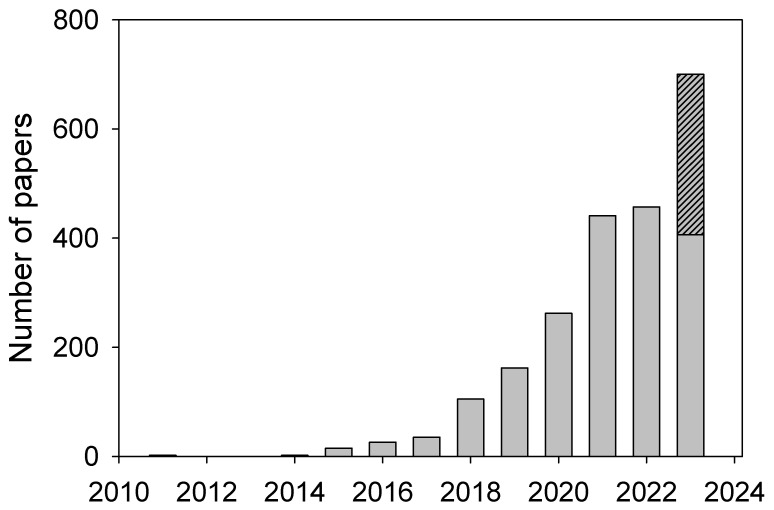
Increase in the number of publications dealing with the different aspects of membraneless organelles. Plot represents the output of a PubMed search for “membraneless organelles” conducted on 9 August 2023. The actual annual publication rate is shown by gray bars, whereas the hatched gray bar shows extrapolated data based on the number of papers published in 2023 by the date of analysis.

## Data Availability

No new data were created or analyzed in this study.
